# Whole-Genome analysis of *Bacillus subtilis* NRCB002 and characterization of its metabolite acetoin as a plant growth stimulant

**DOI:** 10.3934/microbiol.2025024

**Published:** 2025-07-21

**Authors:** Yu Song, Rongjun Yin, Hui Shen, Xin Tao, Linmei Li, Nan Gao

**Affiliations:** School of Biotechnology and Pharmaceutical Engineering, Nanjing Tech University, Nanjing 211816, China

**Keywords:** *Bacillus subtilis*, whole-genome sequencing, acetoin, in-silico analysis, soybean

## Abstract

Plant growth-promoting rhizobacteria (PGPR) are instrumental in enhancing crop productivity and resilience to stress. In this study, we characterized *Bacillus subtilis* subsp. *subtilis* NRCB002, a PGPR strain isolated from the rice rhizosphere, using genomic and functional analyses. Whole-genome sequencing revealed a circular chromosome of 4,211,270 base pairs with a GC content of 43.51%, encoding genes associated with environmental adaptation, such as antimicrobial resistance, and PGPR-related traits, including the biosynthesis of indole-3-acetic acid. The annotation of key metabolic pathways for acetoin production aligns with its observed role in promoting plant growth. Pot experiments demonstrated that optimal acetoin concentrations significantly enhanced the development of soybean seedlings. These findings elucidate the genetic basis of NRCB002's beneficial traits and underscore its potential for agricultural application.

## 1. Introduction

The intensive application of chemical fertilizers and pesticides in modern agriculture has raised obvious environmental and agronomic concerns, including soil degradation, proliferation of soil-borne pathogens, disruption of agroecosystem balance, biodiversity loss, and pollution of aquatic/atmospheric systems [1]. As a sustainable alternative, microbial biofertilizers have emerged as eco-friendly solutions that enhance soil fertility while mitigating environmental impacts [2],[3]. Plant growth-promoting rhizobacteria (PGPR) serve as the predominant microbial inoculants in biofertilizer formulations. Notably, *Bacillus* species have gained prominence due to their dual functionality in plant growth enhancement and stress mitigation through direct and indirect mechanisms [4]–[6]. A thorough comprehension of the genomic characteristics of *Bacillus* species enhances the potential for strain improvement and its applications in agriculture.

Furthermore, *Bacillus* may produce acetoin (3-hydroxy-2-butanone), a volatile organic compound that enhances host plant growth and induces systemic resistance against abiotic stresses [7]. Empirical evidence confirms these growth-promoting effects across plant species, including tomato, lettuce, and *Arabidopsis thalian* [8]–[10]. Initial investigations by Ryu et al. [11] revealed that *B. subtilis* GB03-derived acetoin significantly enhanced *Arabidopsis* biomass and leaf expansion under axenic culture conditions, with subsequent studies demonstrating acetoin's (1 g·L^–1^) efficacy in improving lettuce seedling development under salt stress (3 g NaCl kg^–1^ soil). [9]. *Bacillus subtilis* subsp. *subtilis* NRCB002 (NRCB002), isolated from the rice rhizosphere, can generate acetoin [12]. This species can stimulate the growth of alfalfa under salt stress and lettuce seedlings under normal conditions [13],[14]. A comprehensive investigation of acetoin's growth-promoting effects across diverse crop species is critically important.

Soybean (*Glycine max* (L.) Merr), a globally leguminous crop, serves as both a vital protein source and industrial feedstock, with its seeds containing high protein and oil content [15],[16]. Here, we aim to elucidate environmental adaptation mechanisms of NRCB002 through whole-genome sequencing and functional annotation and to demonstrate the dual role of acetoin in enhancing IAA biosynthesis of NRCB002 and soybean seedling growth.

## 2. Materials and methods

### 2.1. Bacterial strain, DNA extraction, whole-genome sequencing, and annotation

NRCB002 was cryopreserved at -80 °C in LB broth containing 20% (v/v) glycerol. A single colony was selected and cultured in LB liquid medium overnight at 37 °C for DNA extraction. The strain has been deposited in the China General Microbiological Culture Collection Center (CGMCC) with the accession number CGMCC No. 17213. DNA extraction, sequencing, and assembly were performed in Suzhou Genewiz Biotechnology Co., Ltd. (Suzhou, China) on both Illumina NovaSeq 6000 and PacBio Sequel II platforms. For genome assembly, HiFi reads were processed with Hifiasm (v0.13-r308) and corrected with Canu (v1.7). The assembly was validated by mapping Illumina reads (BWA-MEM v0.7.17) and polished iteratively (Pilon v1.24) to improve accuracy. Protein-coding genes were functionally annotated using Prokka (v1.14.6) and Bakta (v1.8.2; database v5.0-Light). Homology-based classification was performed against the Clusters of Orthologous Groups (COG) database (2023 release). For Pathway analysis, we utilized KEGG and Gene Ontology (GO) annotations through the DAVID platform (v6.8).

### 2.2. Genome mapping and resistance gene analysis

Genome visualization was performed using Proksee (https://proksee.ca/) to generate circular genome maps with annotated features, including protein-coding genes, tRNAs, rRNAs, and GC content distribution. Antibiotic resistance genes were predicted through comparative analysis against the Comprehensive Antibiotic Resistance Database (CARD, version 6.0.3).

### 2.3. Effects of acetoin on indole-3-acetic acid production by NRCB002

A single NRCB002 colony was inoculated into 10 mL LB broth and grown overnight at 30 °C with shaking (200 rpm) to prepare the seed culture.

IAA production was quantified by inoculating seed culture (2% v/v) into 10 mL R2A medium containing different acetoin concentrations (0, 0.01, 0.1, 1, and 10 g L^–1^), with three replicates per treatment. After 48 h incubation at 30 °C (200 rpm), bacterial suspensions were centrifuged (6,000 rpm, 5 min). For colorimetric analysis, 2 mL supernatant was mixed with Salkowski reagent [17] and incubated in the dark (37 °C, 1 h). OD_530_ was measured [18], using uninoculated R2A medium as a blank. IAA concentrations were determined from a standard curve.

### 2.4. Effects of acetoin on soybean seedling growth

Pot experiments were performed using five solutions (0, 0.1, 0.3, 0.5, and 1 g L^−1^ acetoin), conducted in a greenhouse controlled at 25 ± 1 °C, with 70% relative humidity, under a 14/10 h (light/dark) photoperiod [19]. Semi-dwarf soybean seeds (Zhonghuang 13) were surface-sterilized with 2.5% sodium hypochlorite for 10 min and washed ten times with deionized water. Seeds were germinated for 7 d in vegetable soil-filled trays under controlled moisture. Surface-sterilized seeds were germinated in vegetable soil-filled trays under controlled moisture. Uniform germinated seedlings were transplanted (two per pot) into 360 mL pots containing 300 g air-dried soil (soil pH: 7.24; and soil organic matter content: 11.0 g kg^–1^). The soybean seedlings were treated with acetoin at 7 and 14 d after transplantation.

In total, there were four replicates of 2 plants each in a complete randomized block design. Soybean seedlings were harvested at 28 days post-sowing, and growth parameters were quantified using established protocols [20].

## 3. Results

### 3.1. Genome assembly and quality assessment of NRCB002

**Figure 1.**
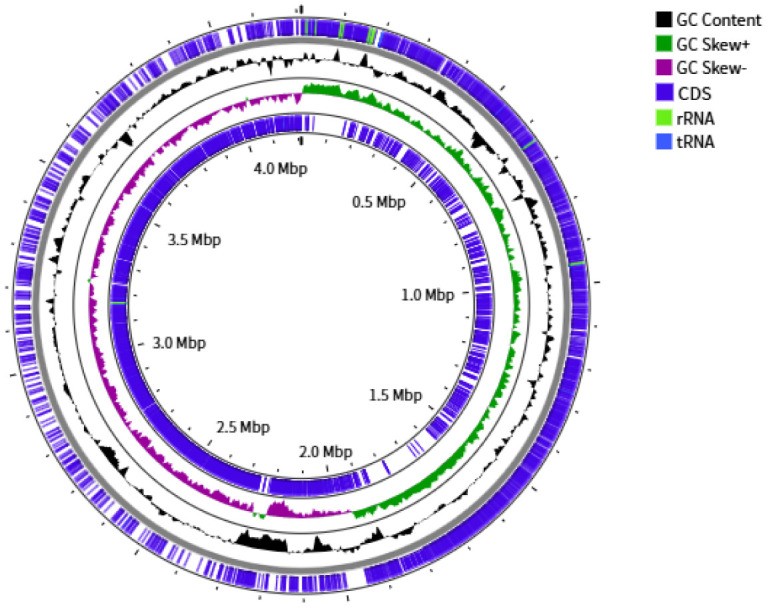
CGView genome map of *Bacillus subtilis* subsp. *subtilis* NRCB002.

Upon genome sequencing and assembly, NRCB002 was given as circular chromosome of 4,211,270 bp (N50 contig size of approximately 4211270 bp) with a GC content of 43.51%. The genome constituted of a total of 4,605 genes, with 30 coding rRNA, 86 tRNAs, and 30 repetitive elements (Figure 1 and Table S1).

### 3.2. KEGG annotation

**Figure 2.**
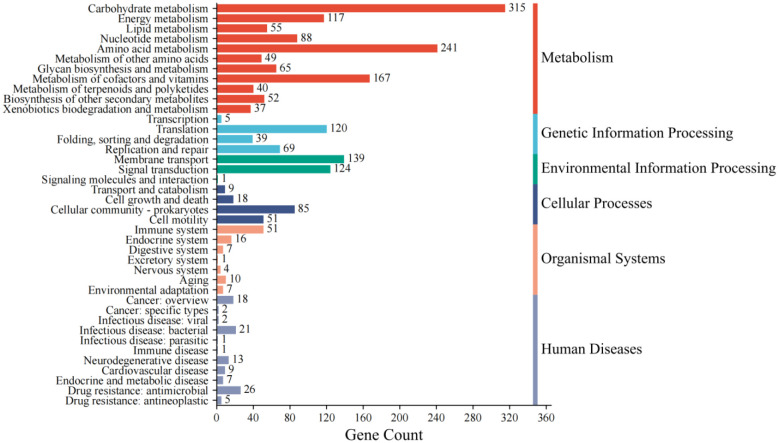
KEGG notes for *Bacillus subtilis* subsp. *subtilis* NRCB002.

KEGG annotation identified six dominant metabolic categories in the NRCB002 genome, with metabolic pathway genes representing the most abundant functional group. Subsequent analysis highlighted robust genetic modules for environmental adaptation (e.g., antibiotic biosynthesis, zeatin production) and stress resilience (glutathione metabolism), correlating with enhanced ecological fitness (Figure 2).

**Figure 3.**
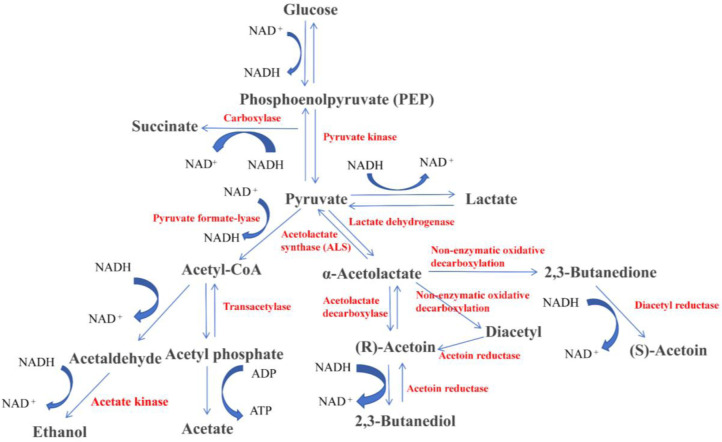
Acetoin metabolism pathway of *Bacillus subtilis* subsp. *subtilis* NRCB002 genome. *Note: Black: Compounds; Red: Enzymes; NAD^+^: Nicotinamide adenine dinucleotide (oxidized form); NADH: Nicotinamide adenine dinucleotide (reduced form); ATP: Adenosine triphosphate; and ADP: Adenosine Diphosphate.

KEGG pathway analysis demonstrated that strain NRCB002 harbors complete biosynthetic pathways for volatile metabolites, including acetoin, poly-γ-glutamic acid, and isovalerate (Figure 3, S1–S2). Acetoin functions as a plant growth promoter and confers broad-spectrum resistance against phytopathogens.

### 3.3. COG annotation

**Figure 4.**
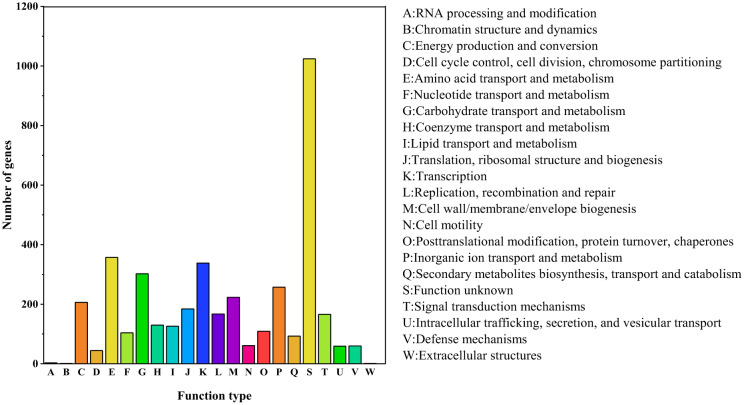
COG database notes for the *Bacillus subtilis* subsp. *subtilis* NRCB002 genome. *Note: The X-axis represents the number of genes, and the Y-axis represents functions of the genes.

A total of 2,991 protein-coding genes were annotated in the COG database [21]. Among these, proteins associated with amino acid transport and metabolism (COG E) represented the most abundant group, followed by those involved in transcription (COG K), carbohydrate transport and metabolism (COG G), and inorganic ion transport and metabolism (COG P). The genomic features of NRCB002 facilitate efficient nutrient assimilation, essential metabolite biosynthesis, and adaptive stress responses, which are consistent with its specialization for complex ecological niches (Figure 4).

### 3.4. GO annotation

**Figure 5.**
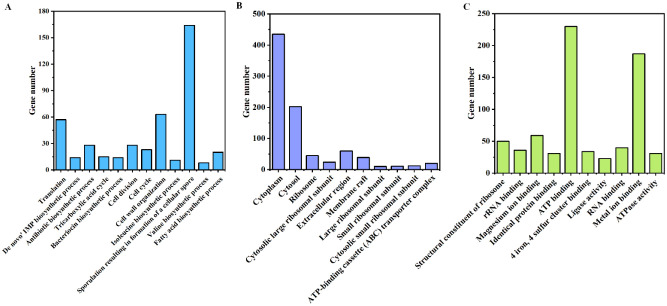
GO annotation and functional classification of *Bacillus subtilis* subsp. *subtilis* NRCB002. *Note: The figure presents the ten highest-ranking pathways based on gene numbers. A. biological processes; B. cellular components; and C. molecular functions.

Through GO annotation of strain NRCB002, a total of 2,183 coding genes were annotated, encompassing three major categories: Molecular function, cellular components, and biological processes (Figure 5). Among the molecular function prediction genes, 230 and 187 genes were associated with "ATP binding" and "metal ion binding", respectively, followed by "magnesium ion binding" (59) and "structural constituent of ribosome" (50). For genes annotated to cellular components, we identified 2322 genes related to "cell", 435 to "cytoplasm", 202 to "cytosol", 60 to "extracellular region", and 45 to "ribosome". Regarding biological processes, 164, 63, 57, 28, and 28 genes were annotated to "sporulation resulting in the formation of a cellular spore", "cell wall organization", "translation", "antibiotic biosynthetic process", and "cell division", respectively.

### 3.5. Analysis of environmentally adaptive genes in NRCB002

The genomic analysis of antimicrobial resistance genes in NRCB002 revealed its environmental flexibility. The genome contains resistance genes for *β*-lactams, vancomycin, and cationic antimicrobial peptides (CAMPs). These genetic characteristics improve the strain's ability to survive in antibiotic-rich settings (Table 1).

**Table 1.** Genes related to antimicrobial substance resistance in *Bacillus subtilis* subsp. *subtilis* NRCB002.

Type	Gene	Product
*β*-lactam resistance	*nagZ*	*β*-N-acetylhexosaminidase
	*ftsl*	3 - Penicillin-binding protein 3 (PBP3)
	*mrcA*	1A - Penicillin-binding protein 1A (PBP1A)
	*oppF, oppD*	Oligopeptide transport ATP-binding protein
	*pbp2A*	2A - Penicillin-binding protein 2A
	*oppA, mppA*	Oligopeptide-binding protein
	*oppB, oppA*	Oligopeptide transport permease
	*penP*	Class A *β*-lactamase
	*oxa*	Class D *β*-lactamase
	*abcA, bmrA*	ABC transporter
Vancomycin resistance	*mraY*	Phospho-N-acetylmuramoyl-pentapeptide-transferase
	*alr*	Alanine racemase
	*ddl*	D-alanine-D-alanine ligase
	*murF*	UDP-N-acetylmuramoyl-tripeptide-D-alanyl-D-alanine ligase
	*murG*	Acetylglucosamine transferase
	*vanY*	D-alanyl-D-alanine carboxypeptidase
	*vanW*	Vancomycin resistance protein
Cationic antimicrobial peptide (CAMP) resistance	*amiABC*	N-acetylmuramoyl-L-alanine amidase N-acetylmuramoyl-L-alanine amidase
	*dltA*	1 - D-alanine-poly(phosphoribitol) ligase subunit 1
	*dltB*	Membrane protein involved in D-alanine export
	*dltD*	D-alanine transporter
	*degP, htrA*	Serine protease
	*dltC*	2 - D-alanine-poly(phosphoribitol) ligase subunit 2
	*mprF, fmtC*	Phosphatidylglycerol lysyltransferase

### 3.6. Effect of acetoin on indole-3-acetic acid production by NRCB002

**Figure 6.**
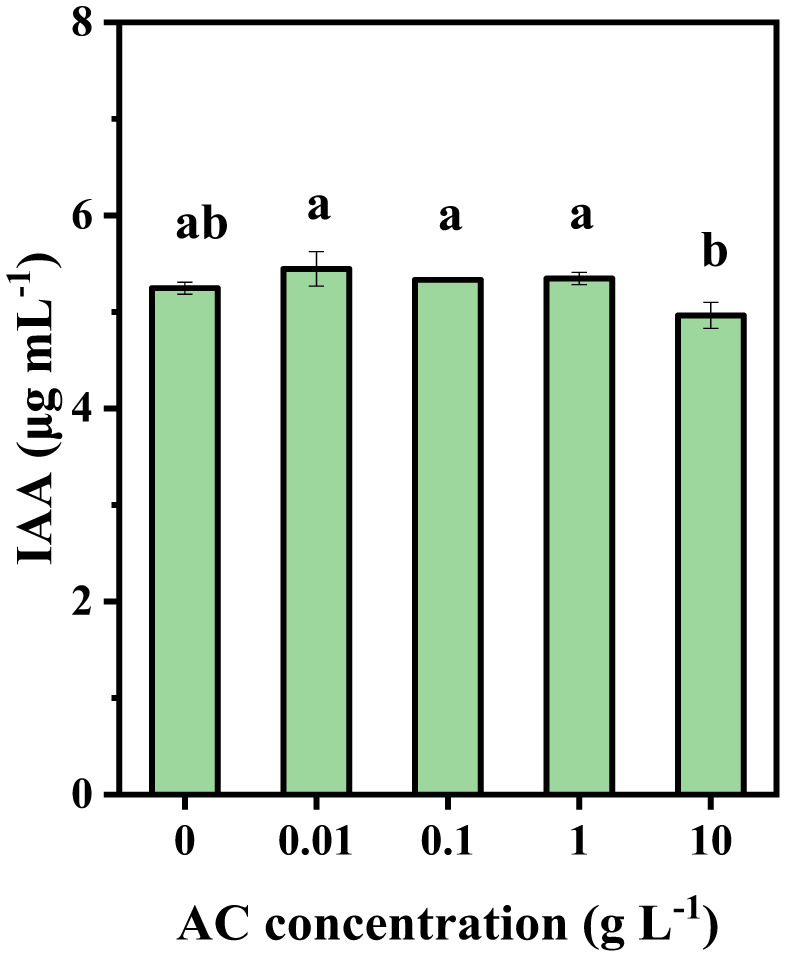
Effect of acetoin (AC) on IAA production by *Bacillus subtilis* subsp. *subtilis* NRCB002. *Note: CK represents the pure water treatment group. Values are presented as means ± standard error, n = 6. Different letters above the bars indicate significant differences between AC concentrations by Duncan's post hoc test (at *p* < 0.05).

Acetoin at 0.01, 0.1, and 1 gL^–1^ markedly increased IAA secretion by strain NRCB002 compared to CK, although IAA production was inhibited at a higher dose (10 g L^–1^) (Figure 6).

### 3.7. Acetoin promotes the growth of soybean seedlings

**Figure 7.**
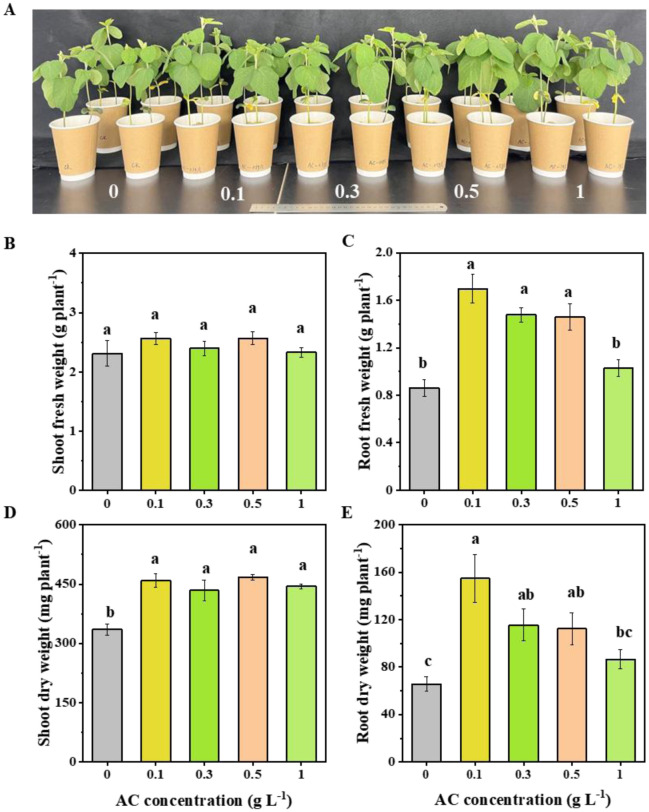
Effects of AC on soybean growth under soil culture conditions. *Note: A phenotype of plants, B shoot fresh weight, C root fresh weight, D shoot dry weight, and E root dry weight. Values are presented as means ± standard error, n = 4 (two seedlings per replicate). Different letters above the bars indicate significant differences between AC concentrations by Duncan's post hoc test (at *p* < 0.05).

When compared to the control, the application of acetoin considerably raised the shoot dry weight of soybean seedlings by 29.6%–37% (Figure 7D). Both root fresh weight (by 96.6%, 71.4%, and 69.2%, respectively) and root dry weight (by 134.8%, 74.2%, and 69.7%, respectively) were significantly increased by treatment with 0.1, 0.3, and 0.5 g L^–1^ acetoin (Figures 7C and E). Furthermore, at 1 g L^–1^ acetoin concentration, acetoin improved root length, root surface, root volume number of root tips, and average root diameter (Table 2).

**Table 2.** Effects of AC on soybean root growth.

AC concentration (g L^–1^)	Root length (cm)	Root surface area (cm^2^)	Root volume (cm^3^)	Number of root tips	Average diameter of root (mm)
0	287.0 ± 29.3c	43.7 ± 8.3c	0.9 ± 0.3c	1447 ± 222b	0.51 ± 0.04b
0.1	874.7 ± 60.2b	321.1 ± 38.6b	31.4 ± 9.7b	4013 ± 282a	0.75 ± 0.01a
0.25	827.9 ± 96.2b	286.6 ± 22.7b	27.3 ± 3.2b	4280 ± 455a	0.74 ± 0.04a
0.5	790.6 ± 38.3b	335.9 ± 28.1b	37.5 ± 3.9b	4076 ± 100a	0.78 ± 0.02a
1	1089.4 ± 82.6a	523.9 ± 63.3a	62.0 ± 7.7a	4178 ± 336a	0.86 ± 0.06a

*Note: Different letters after the values of the same column mean significant differences between treatments by Duncan's post hoc tests (at *p* < 0.05).

## 4. Discussion

*B. subtilis* is a well-studied PGPR that serves as the principal microbial source for the creation of biofertilizer formulations [22]. The processes of genome sequencing and functional annotation are instrumental in elucidating the functional capabilities of microorganisms. The complete genome of NRCB002, comprising 4,211,270 base pairs, was reconstructed utilizing a hybrid sequencing approach that combines PacBio and Illumina technologies (Table S1). This genome size is comparable to that of strain Bbv57, which measures 4,302,465 base pairs [23]. Furthermore, the genomic sequence data of NRCB002 could be compared and functionally annotated through GO, COG, and KEGG databases, which could predict a large amount of iron ion binding, glutathione metabolism, amino acid transport and metabolism, biosynthesis of secondary metabolites, and carbohydrate transport and metabolism (Figure 2, 4 and 5). This metabolic plasticity enables the strain to efficiently acquire nutrients in complicated soil-rhizosphere conditions while biosynthesizing a wide range of bioactive chemicals, which jointly promote plant development and induce systemic resistance [24],[25]. Furthermore, NRCB002 has unique *β*-lactam and vancomycin resistance genes (Table 1) compared to *Bacillus amyloliquefaciens* FZB42, potentially enhancing its ecological competitiveness [26].

NRCB002 demonstrates high-yield production of the volatile metabolite acetoin, which effectively promotes plant growth. Rebuilding NRCB002's metabolic pathway was made possible by the identification of important acetoin biosynthesis genes (*alsS*, *alsD*) [26] by genomic analysis (Figure 3). Researchers have demonstrated that *Bacillus subtilis* BCT9 enhances lettuce growth by secreting modest levels of acetoin [27]. Similarly, *Bacillus velezensis* secretes acetoin, which induces *Arabidopsis* to activate critical antioxidant enzymes, hence increasing *Arabidopsis* growth [28]. Unlike prior investigations, we systematically explore how acetoin concentrations modify IAA production in NRCB002 to improve bacterial growth while determining the best acetoin dosage for promoting soybean seedling growth. Acetoin (0.01 g L^–1^) can increase NRCB002's capacity to generate IAA (Figure 6). The dry weight of the aboveground and underground portions of soybean seedlings was gradually increased by lower concentrations (0.1–0.5 g L^–1^), but the application of 1 g L^–1^ markedly enhanced root growth (Figure 6 and Table 2).

## 5. Conclusion

The genomic analysis of NRCB002 revealed functional gene clusters involved in secondary metabolite biosynthesis, vitamin metabolism, antibiotic generation, and glutathione metabolism. The genome also contained several antibiotic resistance genes, including those that conferred resistance to CAMPs and vancomycin, indicating environmental adaptation. NRCB002-produced acetoin can greatly boost IAA synthesis by the bacterial strain and soybean seedlings growth in a concentration-dependent manner.


