# Supplementary material for: Whole-Genome analysis of Bacillus subtilis NRCB002 and characterization of its metabolite acetoin as a plant growth stimulant
Source: AIMS Microbiol. 2025 Jul 21;11(3):574–87. doi: 10.3934/microbiol.2025024 (PMC12511955; doi:10.3934/microbiol.2025024)
Supplement: Supplementary file 1 [file microbiol-11-03-024-s001.pdf]

AIMS Microbiology, 11(3): 574–587.
DOI: 10.3934/microbiol.2025024
Received: 15 May 2025
Revised: 30 June 2025
Accepted: 08 July 2025
Published: 21 July 2025

https://www.aimspress.com/journal/microbiology

Research article

Whole-Genome analysis of *Bacillus subtilis* NRCB002 and characterization of its metabolite acetoin as a plant growth stimulant

Yu Song, Rongjun Yin, Hui Shen, Xin Tao, Linmei Li and Nan Gao*

School of Biotechnology and Pharmaceutical Engineering, Nanjing Tech University, Nanjing 211816, China

* **Correspondence:** Email: ngao@njtech.edu.cn.

Supplmentary

Reagent Preparation:
Salkowski Reagent Preparation: Mix 50 mL of 35% (w/v) $HClO_4$ with 1 mL of 0.5 M $FeCl_3$ solution. Store protected from light until use.

Table S1. Genome assembly information of *Bacillus subtilis* subsp. *subtilis* NRCB002.

Sample	NRCB002
Bases	4211270
rRNA	30
tRNA	86
Repetitive sequences	30
N50	4211270
Mean depth	74.06
(C+G) %	43.51%
Coverage (%)	100%

Table S2. GO database notes for the *Bacillus subtilis* subsp. *subtilis* NRCB002 genome.

GO identifier	Term	Gene number	Category
GO:0006412	translation	57	BP
GO:0006189	de novo' IMP biosynthetic process	14	BP
GO:0017000	antibiotic biosynthetic process	28	BP
GO:0006099	tricarboxylic acid cycle	15	BP
GO:0030152	bacteriocin biosynthetic process	14	BP
GO:0051301	cell division	28	BP
GO:0007049	cell cycle	23	BP
GO:0071555	cell wall organization	63	BP
GO:0009097	isoleucine biosynthetic process	11	BP
GO:0030435	sporulation resulting in formation of a cellular spore	164	BP
GO:0009099	valine biosynthetic process	8	BP
GO:0006633	fatty acid biosynthetic process	20	BP
GO:0005737	cytoplasm	435	CC
GO:0005829	cytosol	202	CC
GO:0005840	ribosome	45	CC
GO:0022625	cytosolic large ribosomal subunit	24	CC
GO:0005576	extracellular region	60	CC
GO:0045121	membrane raft	39	CC
GO:0015934	large ribosomal subunit	10	CC
GO:0015935	small ribosomal subunit	11	CC
GO:0022627	cytosolic small ribosomal subunit	12	CC
GO:0043190	ATP-binding cassette (ABC) transporter complex	20	CC
GO:0003735	structural constituent of ribosome	50	MF
GO:0019843	rRNA binding	36	MF
GO:0000287	magnesium ion binding	59	MF
GO:0042802	identical protein binding	31	MF
GO:0005524	ATP binding	230	MF
GO:0031177	phosphopantetheine binding	13	MF
GO:0051539	4 iron, 4 sulfur cluster binding	34	MF
GO:0000049	tRNA binding	24	MF
GO:0005506	iron ion binding	19	MF
GO:0016874	ligase activity	23	MF
GO:0004315	3-oxoacyl-[acyl-carrier-protein] synthase activity	9	MF
GO:0003723	RNA binding	40	MF
GO:0046872	metal ion binding	187	MF
GO:0030145	manganese ion binding	15	MF
GO:0050661	NADP binding	19	MF
GO:0016887	ATPase activity	31	MF
GO:0004252	serine-type endopeptidase activity	19	MF
GO:0009002	serine-type D-Ala-D-Ala carboxypeptidase activity	11	MF
GO:0046933	proton-transporting ATP synthase activity, rotational mechanism	12	MF
GO:0003924	GTPase activity	57	BP

Table S3. Effects of acetoin on tomato shoots growth.

AC concentration L^{-1}) (g	SPAD	Shoot height (cm)	Stem diameter (mm)	Leaf area (mm^2)	Leaf perimeter (mm)
0	40.8 ±2.0b	15.0 ±1.7a	2.86 ±0.15a	285.0 ±46.2a	235.9 ±9.4a
0.1	43.0 ±0.5ab	15.5 ±0.9a	2.81 ±0.07a	337.7 ±51.4a	300.9 ±40.5a
0.3	43.2 ±1.0ab	15.1 ±0.7a	2.87 ±0.11a	385.8 ±32.2a	243.2 ±13.1a
0.5	44.9 ±0.6a	15.6 ±0.7a	2.85 ±0.09a	459.1 ±88.5a	268.8 ±25.0a
1	44.4 ±1.2ab	17.8 ±1.4a	2.95 ±0.05a	440.1 ±38.1a	250.4 ±27.1a

*Note: Different letters after the values of the same column mean significant differences between treatments by Duncan's post hoc tests (at $p < 0.05$).

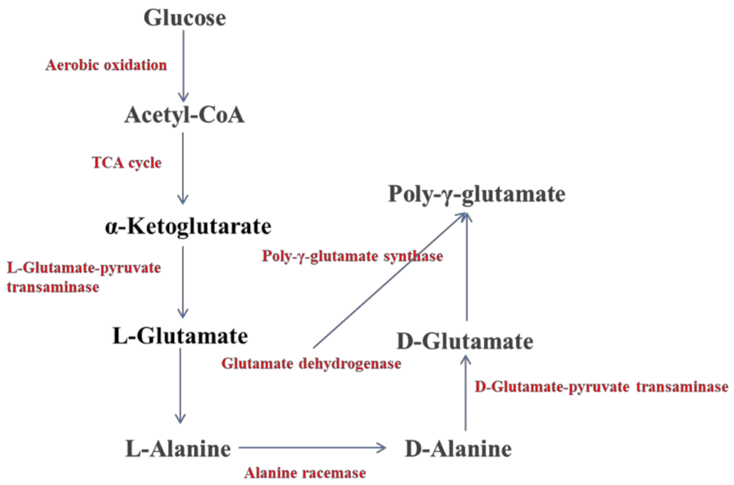

Figure S1. poly-γ-glutamic acid metabolism pathway of *Bacillus subtilis* subsp. *Subtilis* NRCB002 genome. *Note: Black: compounds; Red: Enzymes; TCA cycle: Tricarboxylic acid cycle.

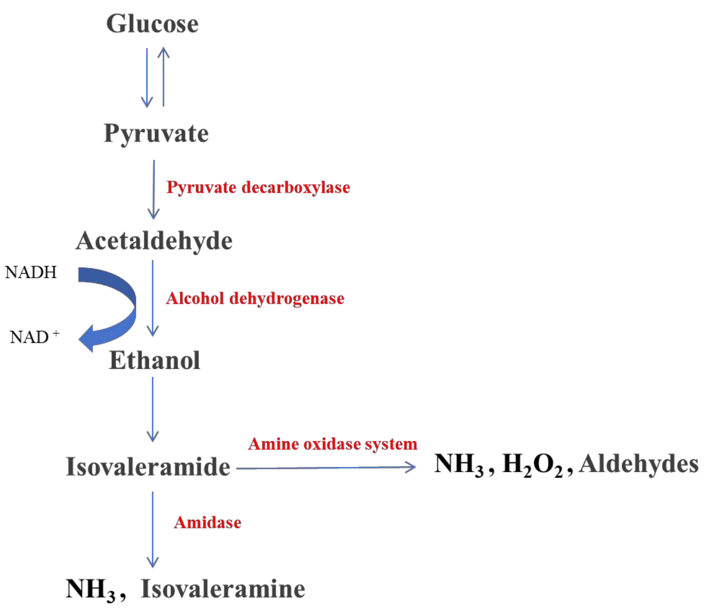

Figure S2. Isovaleramide metabolism pathway of *Bacillus subtilis* subsp. *subtilis* NRCB002 genome. *Note: Black: compounds; Red: Enzymes; NAD^+: Nicotinamide adenine dinucleotide (oxidized form); NADH: Nicotinamide adenine dinucleotide (reduced form); H_2O_2: Hydrogen peroxide.

AIMS Press

© 2025 the Author(s), licensee AIMS Press. This is an open access article distributed under the terms of the Creative Commons Attribution License (https://creativecommons.org/licenses/by/4.0)
